# High-Precision Regulation of Nano-Grating Linewidth Based on ALD

**DOI:** 10.3390/mi13070995

**Published:** 2022-06-24

**Authors:** Yaxin Zhang, Chenying Wang, Weixuan Jing, Song Wang, Yujing Zhang, Liangliang Zhang, Yijun Zhang, Nan Zhu, Yunxiang Wang, Yifan Zhao, Qijing Lin, Zhuangde Jiang

**Affiliations:** 1 State Key Laboratory for Manufacturing Systems Engineering, International Joint Laboratory for Micro/Nano Manufacturing and Measurement Technologies, Xi’an Jiaotong University, Xi’an 710049, China; zhangyaxin@stu.xjtu.edu.cn (Y.Z.); wangsong2015@stu.xjtu.edu.cn (S.W.); zhang2020@stu.xjtu.edu.cn (Y.Z.); zhangliangliang@mail.xjtu.edu.cn (L.Z.); nan.zhu@xjtu.edu.cn (N.Z.); qjlin2015@mail.xjtu.edu.cn (Q.L.); zdjiang@xjtu.edu.cn (Z.J.); 2 School of Mechanical Engineering, Xi’an Jiaotong University, Xi’an 710049, China; 3 Collaborative Innovation Center of High-End Manufacturing, Xi’an Jiaotong University, Xi’an 710049, China; 4 Electronic Materials Research Laboratory, Key Laboratory of the Ministry of Education & International Center for Dielectric Research, School of Electronic Science and Engineering, Xi’an Jiaotong University, Xi’an 710049, China; zhangyj518@mail.xjtu.edu.cn; 5 Suzhou Institute of Metrology, Suzhou 215128, China; wangyx@szjl.com.cn

**Keywords:** atomic layer deposition (ALD), linewidth regulation, micro- and nano-metrology, one-dimensional nano-grating standard

## Abstract

A nano-grating standard with accurate linewidth can not only calibrate the magnification of nano-measurement instruments, but can also enable comparison of linewidths. Unfortunately, it is still a challenging task to control the linewidth of nano-grating standards. Accordingly, in this paper, atomic layer deposition (ALD) was used to regulate the linewidth of the one-dimensional grating standards with a pitch of 1000 nm, fabricated by electron beam lithography (EBL). The standards were measured using an atomic force microscope (AFM) before and after ALD, and the linewidth and pitch of the grating were calculated through the gravity center method. The obtained results prove that the width of a single grating line in the standard can be regulated with great uniformity by precisely utilizing ALD. Meanwhile, the proposed method does not affect the pitch of grating, and the measurement uncertainty of standards is less than 0.16% of the pitch, thereby demonstrating a high surface quality and calibration reliability of the standards, and realizing the integration of linewidth and pitch calibration functions. Moreover, the precise and controllable fabrication method of the micro-nano periodic structure based on ALD technology has many potential applications in the fields of optoelectronic devices and biosensors.

## 1. Introduction

With the rapid advances in nanofabrication technology, the minimum gate length scales of transistors have been reduced to sub-10 nm [1,2,3,4,5], so they require precise geometric measurements, which in turn induces high demands on the accuracy of nano-measurement instruments. Therefore, it is necessary to develop nano-standards with traceability to calibrate the nano-measurement instruments to ensure the accuracy of characterization in nanofabrication, and accordingly, improve the performance of integrated circuits. In this regard, one-dimensional nano-grating standards, as one type of the important nanometric standards, are mainly used to calibrate the magnification of nano-measurement instruments. Correspondingly, a large number of research institutions and companies have developed a series of one-dimensional (1D) micro- and nano-grating standards [6,7,8,9,10,11,12,13]. Nevertheless, most nano-grating standards have a single function which provides reliable pitch calibration values. With such standards, the controllability and uniformity of the linewidth cannot be guaranteed, hence the linewidth cannot be calibrated. In addition, a grating with a constant duty cycle cannot simultaneously match the different requirements of measurement instruments with different calibration principles, for the optimal duty cycle. That is to say, the efficiency and accuracy of the calibration can be improved by making the linewidth or duty cycle of the nano-grating standard controllable.

Micro- and nano-fabrication processes such as electron beam lithography (EBL), focused ion beam (FIB) and extreme ultra-violet (EUV) are commonly used for manufacturing micro- and nano-structures. Most of the grating standards with pitch ranging between 100 and 4000 nm and fabricated using EBL have good periodicity. However, the linewidths fabricated by the EBL process have certain randomness due to the inevitable proximity effect and the instability of the current [14,15,16]. Therefore, despite using the same parameters, producing the same linewidth each time cannot be guaranteed. The FIB technology is a direct patterning process without a photoresist and includes several basic principles such as milling and deposition [17]. The edge and surface roughness of grating structures obtained by milling is large due to material redeposition [18]. While the structures obtained by FIB deposition have more uniform surface topography, the thickness of the deposited metal will change the designed linewidths at the same time [19]. EUV requires a mask, so the graphic size cannot be flexibly adjusted in time according to the experimental results [20]. Furthermore, none of the above processes can repatch the linewidth again after fabrication. In summary, it is not easy to precisely regulate the linewidth or duty cycle of each grating line, and likewise, it is more challenging to ensure the linewidth accuracy of nano-grating standards.

Atomic layer deposition (ALD) can precisely grow thin films of controlled thickness (from a few to tens of nanometers) on the underlying three-dimensional (3D) structures, with high accuracy, uniformity and consistency. The technique of depositing 3D conformal films on periodic structures using ALD has also been demonstrated in several papers [21,22,23,24,25]. To develop multifunctional grating standards with controllable linewidth and pitch, 1D nano-grating standards with a theoretical pitch of 1000 nm were fabricated in this paper using EBL, and the linewidth of shaped nano-grating was regulated by ALD. In addition, the linewidth and pitch of the 1D grating structure were measured and evaluated by an atomic force microscope (AFM) and a scanning electron microscope (SEM), which validated the feasibility and excellent performance of precise linewidth regulation via ALD, and demonstrated the high surface quality, calibration reliability, and measurement consistency of the standards.

## 2. Design and Fabrication

### 2.1. Structural Design

**Figure 1:**
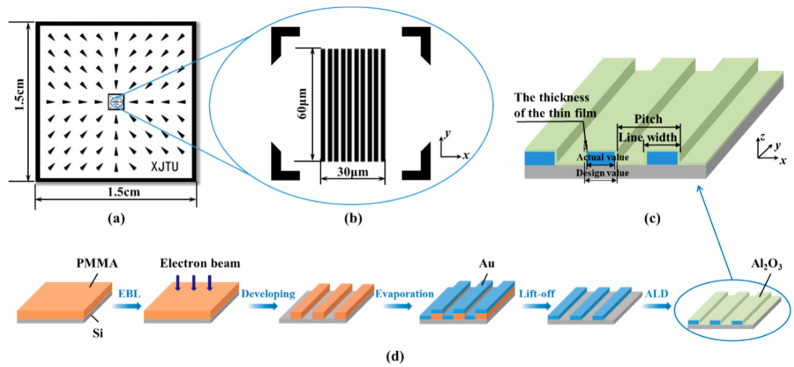
(**a**) Schematic of the guidance area of 1D grating standard. (**b**) Schematic of the calibration area of 1D grating standard. (**c**) Schematic of the linewidth regulation by ALD. (**d**) Schematic of the fabrication process of 1D grating standard.

The 1D grating standard presented in this study is a 1.5 cm × 1.5 cm chip, whose surface structure mainly includes the calibration area and the guidance area, as shown in Figure 1a,b. The calibration area is a 1000 nm pitch 1D grating structure with an overall size of 30 μm × 60 μm (Figure 1b). Notably, the size of the 1D grating structure is very small, only 0.4% of the size of whole sample. It is difficult for the users to position the grating correctly when calibrating; therefore, a guidance area is designed at the periphery of the calibration area (Figure 1a). This area consists of a multi-level marker pattern pointing towards the center of the sample, which helps the users to identify the placement orientation of the sample and locate the calibration area rapidly, hence improving calibration efficiency greatly.

### 2.2. Materials and Fabrication

The substrate material of the standard is Si(100) wafer, while the grating material is Cr and Au. The Au with well wear resistance, stability and conductivity can be used to calibrate the measurement instruments that require the conductivity of the material, such as SEM, and will have an excellent contrast with the substrate. The material for the linewidth regulation is an amorphous Al_2_O_3_ film deposited by ALD. The film growth mode corresponds to a self-limiting chemical reaction between the chemical vapor-phase precursors and the substrate surface, in the ALD process. It is worthwhile to note that the number of reacting precursors on the surface does not increase further, when the surface chemisorption reaches the saturation. As a result, ALD controls the film growth accurately by adding single atomic layers one by one until the film thickness reaches a preset value, thereby ensuring 100% uniformity and conformity of the film. Therefore, a 1D nano-grating standard with controlled linewidth can be produced by depositing an Al_2_O_3_ film on the surface of the grating structure with a thickness that is half the deviation of the linewidth (Figure 1c).

All the experiments were performed in a class 1000 clean room with a constant temperature of (25 ± 1) °C. The patterns of sizes 10–200 μm in the guidance area were fabricated by conventional micro-fabrication processes including ultraviolet lithography and lift-off process [26]. The specific fabrication and regulation process of the 1D grating structure is demonstrated in Figure 1d. The sample was cleaned sequentially in acetone, isopropanol (IPA) and deionized water. After that, a layer of polymethyl methacrylate (PMMA) photoresist AR-P 679 with a thickness of 100 nm was spin-coated on the substrate at 2000 rpm and baked for 2 min at 150 °C on a hot plate. Then, the one-dimensional grating structure pattern was exposed on the photoresist layer using EBL (CABL-9000C, Crestec, Hamamatsu, Japan). After the EBL process, the sample was developed in a mixture of methyl isobutyl ketone (MIBK) and IPA (1:3) for 1 min at 25 °C. Progressively, 5 nm Cr and 25 nm Au films were evaporated (TF500, Hind High Vacuum, Crawley, United Kingdom) on the sample, followed by the removal of remaining photoresist in dioxolane solution for 10 min at 25 °C. The sample was then cleaned with acetone, IPA and deionized water for 5 min each. Next, the grating structure was measured by AFM (INNOVA, Bruker, Karlsruhe, Germany) and the deviation between the designed dimension of linewidth and the actual fabricated dimension was calculated. Finally, the three-dimensional amorphous Al_2_O_3_ thin film was grown on the grating surface by ALD (R-200, Picosun, Masala, Finland). All the specific parameters of the ALD process have been described in our previous works [26].

To study the controllability of the modulated linewidth by ALD, three 1D grating standards with a pitch of 1000 nm, named A, B and C, were fabricated in the experiment. By depositing Al_2_O_3_ films of 5, 10, and 15 nm thickness on the surfaces of samples A, B, and C, respectively, each side of the grating lines is expected to widen by 5, 10, and 15 nm, consequently increasing the width of grating lines by 10, 20, and 30 nm.

### 2.3. Measuremnt

The 1D nano-grating standards were measured by the AFM in the tapping mode. The scanning range was selected at the center of the grating with a size of 10 μm × 10 μm, and the number of sampling points was selected to be 256. The data measured by AFM are inevitably interspersed with some low-frequency noise signals coupled with the profile data, which can lead to bowing distortion of the measured image. Further, there exists a certain cosine error between the sample and the measurement instrument, when the sample is placed on the measurement bench. To better extract the linewidth and pitch data of the standard, linear interpolation, filtering and cosine error correction were applied to the original measurement data, to effectively reduce the tilt, bowing and other low-frequency noise, while preserving the real surface topography of the standard.

## 3. Results and Discussion

### 3.1. Results of Regulation

**Figure 2:**
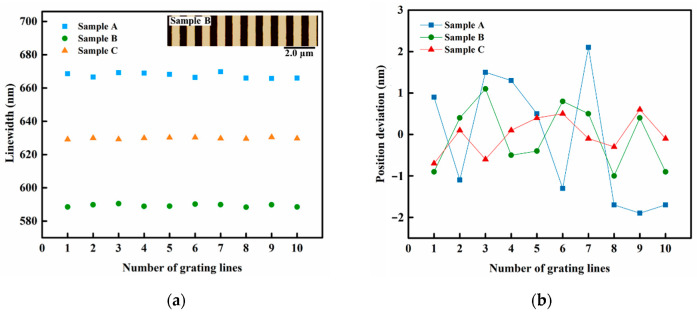
(**a**) Calculated linewidths of the 10 grating lines of samples A, B and C, after ALD. (**b**) The position deviation curves of the calculated linewidths.

Firstly, in order to evaluate the linewidth uniformity of each grating line, ten grating lines in the standard were selected and their linewidths were calculated and analyzed. A length of L = 2000 nm was intercepted from each grating line, where 20 positions were chosen uniformly from top to bottom. The linewidth at each position of the grating line was the difference between the threshold line calculated by the gravity center method [27] and the two intersection points generated by left and right edges of grating line. The results of the linewidth of 10 grating lines for the three samples after ALD are shown in Figure 2a, while the position deviation curves of the linewidths are shown in Figure 2b. The inset in Figure 2a shows the AFM image of sample B after ALD. As evident from the figure, the 1D grating standard possesses well-distributed grating lines and excellent parallelism. However, there are a few particles or defects introduced by the tensile stress on Au with high ductility, during the lift-off process. It is shown in Figure 2a that the linewidths of the ten grating lines of each sample are close to each other with a slight degree of fluctuation. The deviation between the linewidths of 10 grating lines of sample A is the largest, but the maximum deviation is still only 2.1 nm (Figure 2b), which accounts for only 0.3% of the linewidth, thereby indicating the uniformity of linewidths of multiple grating lines in this structure. Hence, the user can select any grating line for the linewidth calibration, which certainly improves the repeatability of the calibration results.

**Figure 3:**
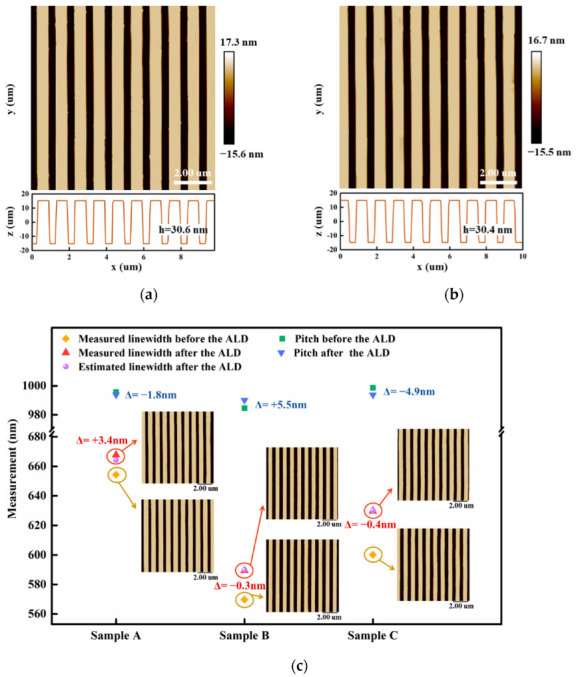
(**a**) The image of sample A before ALD by AFM. (**b**) The image of sample A after ALD by AFM. (**c**) Comparison of linewidths and pitches of samples A, B, and C, before and after ALD.

The average value of the linewidths of 10 grating lines was taken as the linewidth calibration value of each sample. The AFM images and comparison of the linewidths of the three samples before and after ALD are shown in Figure 3. It is shown in Figure 3a,b that the height of the grating was not changed and only the grating lines were widened since ALD was growing the film simultaneously in the 3D direction. Here, the actual increase in the linewidths of three samples was 13.4, 19.6, and 29.7 nm, respectively. Evidently, the actual increase in the linewidths of samples B and C was close to the estimated value, while the actual increase in the linewidth of sample A deviated from the expected increment by 3.4 nm. This is because the linewidth uniformity of sample A is worse than the other two samples, as shown in Figure 2b. Further, the line edge roughness (LER) of the three samples was calculated according to Equation (1) [28] as LER_A_ = 18.9 nm, LER_B_ = 16.4 nm, and LER_C_ = 15.9 nm, respectively. Thus, the final evaluation results are likely to be disturbed by many parameters such as the selection of linewidth evaluation position, quality of grating line edge, and linewidth evaluation algorithm when measuring and calculating the linewidth of this standard.

(1) $$\{\begin{array}{l} \begin{array}{c} \bar{x}=\frac{\left(\sum_{i=1}^{N}x_{i}\right)}{N} \end{array}\\ LER=3\sigma =3\sqrt{\frac{\sum_{i=1}^{N}{\left(x_{i}-\bar{x}\right)}^{2}}{N-1}} \end{array}\;$$

where $\bar{x}$ is the average edge of grating line, *x_i_* is the intersection of threshold line and grating profile, *N* is the number of intersections, and *σ* is the standard deviation of line edge.

The results here reveal that the linewidth of the standard can be regulated by the ALD process, nevertheless there are some certain requirements for the standard fabrication and measurement process: (1) The regulation scheme is unidirectional since ALD can only increase the linewidth of the convex structures or decrease the linewidth of the concave structures. Thus, it is necessary to confirm the desired range of linewidths when processing the grating structure with EBL, according to the type of grating structure (convex or concave). (2) The effect of regulation is related to the linewidth uniformity and the edge straightness of grating line. The better the linewidth uniformity and edge straightness of grating line, the better the regulation performance. (3) The linewidth evaluation algorithm can be further optimized by filtering out various noises as well as the disturbances of particles at the edge of the line. The actual increase in the calculated linewidth in this case will be more reliable and can be fed back to the ALD process, to further improve the experimental parameters and form a closed-loop control.

**Figure 4:**
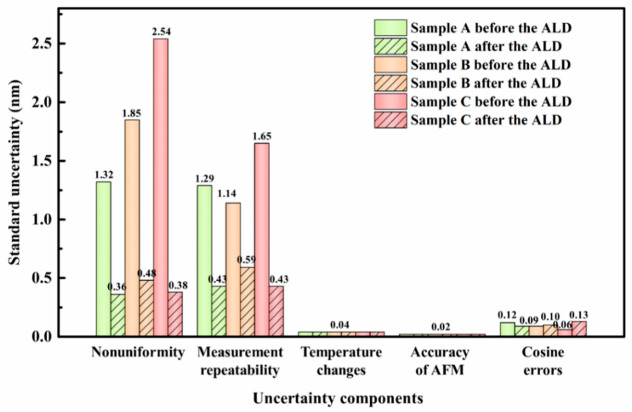
The standard uncertainty of major uncertainty components of samples A, B, and C, before and after ALD.

While aiming to compare the changes in the pitch before and after the regulation, all raw data of 10 scanning lines were obtained uniformly from top to bottom along the y-direction within the scanning range of the sample, and the average pitch of each sample was calculated by the gravity center method. Meanwhile, the measurement uncertainty of each sample was evaluated according to the International Bureau of Weights and Measures (BIPM), the International Electrotechnical Commission (IEC), the International Federation of Clinical Chemistry and Laboratory Medicine (IFCC), the International Organization for Standardization (ISO), the International Union of Pure and Applied Chemistry (IUPAC), the International Union of Pure and Applied Physics (IUPAP), and the International Organization of Legal Metrology (OIML)-1993 Guide to the Expression of Uncertainty in Measurement [29], and the corresponding results are provided in Figure 3 and Figure 4, and Table 1. The average pitch of three samples A, B and C changed by 1.8, 5.5 and 4.9 nm, respectively, before and after ALD. Theoretically, ALD should not change the pitch of the grating, and a small variation in the actual results may be caused by the fact that the quality of ALD depends on the quality of the substrate surface [30]. Hence, when there are large raised particles of several nanometers in size at the edge of grating lines, the surface of such raised particles is uniformly covered with a layer of Al_2_O_3_ film after ALD due to the three-dimensional conformal property of ALD. The shape of the particles is still retained, which would change the line width of the grating and affect the accuracy of the calculated data in turn. Here, the change in the pitch is small, where the maximum variation is only about 0.5% of the average pitch, which still indicates that the Al_2_O_3_ films deposited by ALD have a great uniformity in terms of film thickness and almost do not change the average pitch of the grating standards. As shown in Table 1, the measurement uncertainty of the standards after ALD is less than 0.16% of the average pitch, thus the calibration reliability is quite satisfactory. Certainly, the uncertainties introduced by the surface uniformity and measurement repeatability of the standards are largely minimized here compared with those before ALD, which validates that the surface quality of standard can be optimized and the measurement uncertainties can be reduced by utilizing ALD.

### 3.2. Application

**Figure 5:**
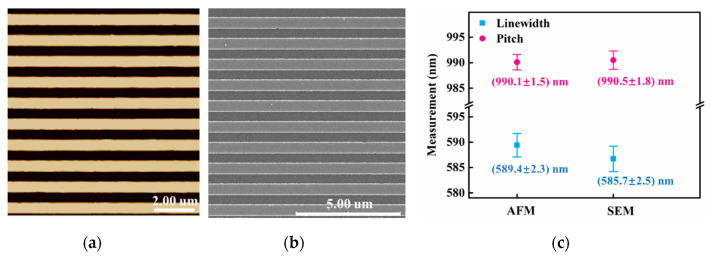
(**a**) The image of sample B after ALD by AFM. (**b**) The image of sample B after ALD by SEM. (**c**) Comparison of the calculation results of linewidth and pitch.

To verify the calibration applicability of the sample obtained from this method in different measurement instruments, the AFM and SEM were used to measure sample B. The data obtained from the two measurement instruments were analyzed to provide a reference for the nano-geometry measurements between different instruments. The linewidth and pitch of sample B were measured by SEM system (SU8010, Hitachi, Tokyo, Japan), calculated by the gravity center method, and then evaluated for the uncertainties. The comparison of the SEM and AFM images, along with the evaluation results, is shown in Figure 5. The linewidth of the standard measured by AFM was evaluated as (589.4 ± 2.8) nm (*k* = 2) while the pitch was evaluated as (990.1 ± 1.5) nm (*k* = 2). On the other hand, the linewidth of the standard measured by SEM was evaluated to be (585.7 ± 3.1) nm (*k* = 2), whereas the pitch was (990.5 ± 1.8) nm (*k* = 2). Clearly, the pitch measured by the both instruments is very close. However, the difference in the linewidths is more obvious.

In this work, the *E_n_* [31] was used to assess the level of agreement between the two measurements, which can be defined using Equation (2). When |*E_n_*| ≤ 1, the consistency of the results is good and acceptable; whereas |*E_n_*| > 1 indicates a poor consistency of the results, which is unacceptable. Based on this criterion, the |*E_n_*|_pitch_ = 0.17 and the |*E_n_*|_linewidth_ = 0.79 were calculated for the considered sample, illustrating high agreement between the pitch and linewidth values obtained using two measurement instruments.

(2) $$E_{n}=\frac{x_{AFM}-x_{SEM}}{\sqrt{U^{2}{}_{AFM}+U^{2}{}_{SEM}}}$$

where *x_AFM_* is the value measured by AFM; *x_SEM_* is the value measured by SEM; *U_AFM_* is the expanded uncertainty of the result measured by AFM; *U_SEM_* is the expanded uncertainty of the result measured by SEM.

Compared with SEM, AFM can measure the 3D surface morphology of the sample more accurately, and the resolution of AFM in the horizontal and vertical directions is close to the atomic scale, hence the measurement uncertainty by AFM is lower. Meanwhile, the measurement uncertainty for SEM is higher, which is mainly introduced by the errors in the image resolution and the variation of electron beam spot diameter. However, the width of AFM probe cannot be neglected while measuring the linewidth, which can induce a spreading effect in the scanning image. As a result, the shape of AFM probe has a significant impact on the linewidth measurement, thereby resulting in a larger linewidth measured by AFM compared to SEM.

On the basis of measurement results, the sample satisfies a cross-comparison of the measurement capabilities of two measurement instruments. Excellent 3D morphology measurements were realized in the AFM, and clear edges of grating lines along with a sharp contrast with the substrate were demonstrated in the SEM. In conclusion, it can be assessed that the consistency level of results obtained by both instruments is superior in this work, based on the *E_n_*. Accordingly, this experiment demonstrates that the samples obtained via precise linewidth regulation based on ALD can be applied to many different types of measurement instruments, where simultaneous calibration of nanoscale pitch and linewidth can be achieved, thereby enhancing calibration efficiency substantially.

## 4. Conclusions

In this work, we have studied the controllable regulation of the linewidth of a 1D grating standard with a pitch of 1000 nm, using ALD. The results reported herein show that the linewidth of the standard can be regulated precisely by utilizing a thin film with controllable thickness and 3D conformal structure, based on the self-limiting layer-by-layer deposition mechanism of ALD. Moreover, the better the edge straightness and linewidth uniformity of the grating line, the better the regulation performance. Evidently, the ALD process can improve the surface uniformity as well as the measurement repeatability of the standard, and restrict the measurement uncertainty of the grating standard below 0.16% of the average pitch, thus potentially guaranteeing the calibration reliability of the standard. Since the thickness of thin film grown by ALD generally does not exceed 100 nm, and the film and substrate bonding will be worse when the film is thicker. That is, the regulation value of this method for linewidth is typically less than 100 nm (film thickness is 50 nm), which requires that the deviation between the designed dimension and the actual fabricated dimension during the patterning process must not be larger than 100 nm. As a result, it is more appropriate for linewidth regulation of the grating standard with a pitch of 100 nm–10 μm.

The results acquired here from the comparisons of linewidth and pitch of the same sample by AFM and SEM are consistent; therefore, the 1D nano-grating standard with controllable pitch and linewidth can be integrated with the calibration function of grating and linewidth. It can be used not only to calibrate the magnification of the measuring instruments, but also to achieve the measurement of critical dimensions of micro- and nano-devices, thereby avoiding the repetitive errors introduced by frequent standard replacement and improving calibration efficiency significantly. Furthermore, it can also be adapted to the measurement requirements of different measurement instruments regarding the duty cycle of standard, easily and efficiently, by simply adjusting the duty cycle of the formed 1D nano-grating standard, which essentially expands the application range of the standard, and economizes the manufacturing expense.

The follow-up work will focus on further reducing the deviation between the actual and estimated regulation values of the linewidth by optimizing the parameters of the ALD process based on the molecular microscopic properties of thin film materials, and realizing precise regulation of the linewidth of 1D grating standards at the sub-nanometer scale.

## Figures and Tables

**Table 1:** The evaluation results of the one-dimensional grating standards.

Sample	A		B		C	
	Before ALD	After ALD	Before ALD	After ALD	Before ALD	After ALD
Average pitch (nm)	995.6	993.8	984.6	990.1	998.7	993.8
Expanded uncertainty (*k* ^1^ = 2) (nm)	3.70	1.14	4.35	1.54	6.06	1.18

^1^*k* is the coverage factor.
